# National Training School for District Midwives

**Published:** 1916-06-24

**Authors:** 

**Affiliations:** 101 Onslow Square, S.W.


*To the Editor of* THE HOSPITAL.

### National Training School for District Midwives.

*To the Editor of* THE HOSPITAL.

SIR,—Will you permit me to draw the attention of your readers to the meeting at the Mansion House on June 27, at 3.30 P.M., at which the Lord Mayor has consented to preside. The object is to assist the National Training School for District Midwives. At all times, but now especially, the work of those who attend a large percentage of women in their confinements and have charge of infants during the first and often critical ten days of their lives is a matter of real national importance. To reduce infant mortality and to secure the greatest possible number of healthy children is a vital need, having regard to the terrible loss of our best manhood, which must gravely affect the future progress of the country.

The meeting will be addressed by Lord Balfour of Burleigh, Sir Dyce Duckworth, M.D., Lady Betty Balfour, and Dr. Barbara Tchaykovsky, and a statement will be made by the hon. secretary, Miss Alice Gregory, on the progress of the Institution.

Tickets can be obtained on application to the Hon. Secretary, British Hospital for Mothers and Babies, Woolwich.—Yours obediently,

(*Signed*) SYDENHAM.
101 Onslow Square, S.W., June 19, 1916.

